# Supplementary material for: Clinical Predictors of Underlying Histologic Activity in Patients with Lupus Nephritis: A Focus on Urinary Soluble CD163
Source: J Clin Med. 2025 Aug 31;14(17):6162. doi: 10.3390/jcm14176162 (PMC12429280; doi:10.3390/jcm14176162)
Supplement: Supplementary file 1 [file jcm-14-06162-s001.zip › jcm-3808471-supplementary.pdf]

Table S1. Correlation of usCD163 with clinical and histological variables.

Variable	*r*	p-value		*r*	p-value
**Age (y)**	0.04	0.77	**Activity Index**	0.47	0.002
**Serum Cr (mg/dl)**	0.24	0.11	Endocapillary hypercellularity	0.47	0.002
**eGFR (ml/min)**	-0.23	0.14	Neutrophils/karyorrhexis	0.51	0.001
**24-h proteinuria (g/day)**	0.7	<0.001	Fibrinoid necrosis	0.27	0.08
**Albumin/creatinine ratio (mg/g)**	0.87	<0.001	Hyaline deposits	0.3	0.05
**Albuminuria (mg/dl)**	0.77	<0.001	Cellular/fibrocellular crescents	0.32	0.03
**Urinary creatinine (mmol/l)**	-0.43	0.004	Interstitial inflammation	0.34	0.02
**Hematuria (cells/µL)**	0.51	<0.001	**Chronicity Index**	0.003	0.98
**ANA (U/mL)**	-0.007	0.96	Total glomerulosclerosis score	-0.003	0.98
**C3 (mg/dl)**	-0.5	0.001	Fibrous crescents	0.08	0.6
**C4 (mg/dl)**	-0.32	0.03	Tubular atrophy	0.04	0.81
			Interstitial fibrosis	0.02	0.91

Abbreviations: *LN, lupus nephritis; eGFR, estimated glomerular filtration rate; patients; ANA, antinuclear antibodies.*

Table S2. Correlation between clinical and histological variables.

Variable	Hematuria		Proteinuria		eGFR		C3		C4		ANA	
	r	**p-value**	***r***	**p-value**	***r***	**p-value**	***r***	**p-value**	***r***	**p-value**	***r***	**p-value**
**Activity Index**	0.28	0.06	0.27	0.08	-0.06	0.68	-0.16	0.31	-0.04	0.76	0.21	0.18
Endocapillary hypercellularity	0.33	0.03	0.41	0.006	-0.12	0.42	-0.2	0.21	0.03	0.84	0.22	0.17
Neutrophils/karyorrhexis	0.32	0.04	0.22	0.16	0.07	0.66	-0.42	0.005	-0.25	0.11	0.11	0.47
Fibrinoid necrosis	0.28	0.06	0.13	0.4	0.07	0.64	-0.14	0.37	-0.06	0.67	-0.08	0.61
Hyaline deposits	0.04	0.8	0.06	0.68	0.02	0.86	-0.25	0.11	-0.21	0.19	0.44	0.004
Cellular/fibrocellular crescents	0.18	0.25	0.31	0.04	-0.12	0.44	0.04	0.79	0.08	0.57	0.11	0.48
Interstitial inflammation	0.21	0.18	0.05	0.73	-0.42	0.005	-0.12	0.42	-0.04	0.79	0.35	0.02
**Chronicity Index**	-0.28	0.06	-0.03	0.82	-0.24	0.11	0.05	0.72	0.04	0.78	0.38	0.01
Total glomerulosclerosis score	-0.25	0.09	0.12	0.42	-0.08	0.61	0	0.99	-0.03	0.82	0.26	0.09
Fibrous crescents	-0.15	0.32	0.04	0.77	-0.11	0.48	0	0.99	-0.05	0.73	-0.13	0.42
Tubular atrophy	-0.25	0.11	-0.08	0.61	-0.36	0.02	0.03	0.83	0.07	0.66	0.39	0.01
Interstitial fibrosis	-0.22	0.16	-0.15	0.33	-0.24	0.11	0.1	0.5	0.06	0.69	0.33	0.03

Abbreviations: *LN, lupus nephritis; eGFR, estimated glomerular filtration rate; ANA, antinuclear antibodies; CRP, C-reactive protein.*

Table S3. Univariate and multivariate logistic regression analysis regarding clinical and histological factors associated with an elevated usCD163.

Variable	Univariate		Multivariate	
	Odds Ratio (95%CI)	p-value	Odds Ratio (95%CI)	p-value
Activity Index (for 1 point)	1.34 (1.11-1.63)	0.003	1.3 (1.05-1.59)	0.01
Chronicity Index (for 1 point)	1.16 (0.89-1.49)	0.25	-	-
Hematuria (for 1 cell/µL)	1.01 (0.99-1.03)	0.17	-	-
24-h proteinuria (for 1 g/day)	1.91 (1.17-3.11)	0.01	1.9 (1.06-3.39)	0.03
eGFR (for 1 ml/min)	0.99 (0.97-1.01)	0.66	-	-
ANA (for 1 U/mL)	1.03 (0.87-1.22)	0.71	-	-
C3 level (for 1 mg/dL)	0.97 (0.95-0.99)	0.03	0.97 (0.93-1.006)	0.1
C4 level (for 1 mg/dL)	0.94 (0.88-1.01)	0.13	-	-

Abbreviations: *eGFR, estimated glomerular filtration rate; ANA, antinuclear antibodies;*
